# Establishment of the mayfly *Cloeon dipterum* as a new model system to investigate insect evolution

**DOI:** 10.1186/s13227-019-0120-y

**Published:** 2019-04-02

**Authors:** Isabel Almudi, Carlos A. Martín-Blanco, Isabel M. García-Fernandez, Adrián López-Catalina, Kristofer Davie, Stein Aerts, Fernando Casares

**Affiliations:** 1 GEM-DMC2 Unit, The CABD (CSIC-UPO-JA), Ctra. de Utrera km 1, 41013 Seville, Spain; 2 Laboratory of Computational Biology, VIB Center for Brain & Disease Research, Herestraat 49, 3000 Louvain, Belgium; 3 Department of Human Genetics, KU Leuven, Oude Markt 13, 3000 Louvain, Belgium

**Keywords:** Evolutionary and developmental biology, Insect evolution, Embryogenesis, Paleoptera, Mayflies, Ephemeroptera, *Cloeon dipterum*, Regeneration

## Abstract

The great capability of insects to adapt to new environments promoted their extraordinary diversification, resulting in the group of Metazoa with the largest number of species distributed worldwide. To understand this enormous diversity, it is essential to investigate lineages that would allow the reconstruction of the early events in the evolution of insects. However, research on insect ecology, physiology, development and evolution has mostly focused on few well-established model species. The key phylogenetic position of mayflies within Paleoptera as the sister group of the rest of winged insects and life history traits of mayflies make them an essential order to understand insect evolution. Here, we describe the establishment of a continuous culture system of the mayfly *Cloeon dipterum* and a series of experimental protocols and omics resources that allow the study of its development and its great regenerative capability. Thus, the establishment of *Cloeon* as an experimental platform paves the way to understand genomic and morphogenetic events that occurred at the origin of winged insects.

**Electronic supplementary material:**

The online version of this article (10.1186/s13227-019-0120-y) contains supplementary material, which is available to authorized users.

## Introduction

Insects are the most diverse group of Metazoa, harbouring the largest number of animal species [1]. Insects comprise more than thirty extant orders distributed worldwide—they are found in all sorts of habitats including marine environments [2, 3]. Despite the fact that other animals populated the land before insects, like chelicerates and myriapods [4–6], the appearance of winged insects meant a complete biological revolution with profound effects on the history of life on earth. The colonisation of the air allowed insects unprecedented dispersal capacities and novel ecological interactions–such as their role as pollinator agents that drove the further coevolution of insects and angiosperms.

Although the impact that the appearance of insects had in the shaping and evolution of, not only their own group, but also other phyla and even kingdoms, our knowledge of insects comes mainly from work on a handful of well-established model species. Among them, *Drosophila melanogaster*, which is one of the best-studied model organisms, is broadly used in multiple fields of research, including the evo-devo field [7–9]. Probably, the second most used insect in evolutionary and developmental studies is *Tribolium castaneum* (Coleoptera), followed by some butterfly and moth (Lepidoptera) species. In addition to these established models, other dipterans with important impact on human health (as vectors transmitting diseases: *Anopheles*, *Glossina*, *Aedes*) and economy (e.g. agricultural pests: *Ceratitis capitata*, *D. suzukii*) have been studied in more detail. Unfortunately, these insect orders are all part of the holometabola group of hexapoda, which appeared relatively recently within the insect phylogeny ([10] and references therein). Some efforts have been made in order to fill the gap in hemimetabola, with the introduction of species such as *Oncopeltus fasciatus* [11], *Blattella germanica* [12] and *Gerris buenoi* or *Rhagovelia antilleana *[13, 14].

This dearth of laboratory models is even more acute in the case of early branching insect groups that correspond to the first representatives of the crucial biological and ecological transitions mentioned above. For instance, key adaptations to terrestrial life such as the development of the extra-embryonic tissues amnion and serosa [15–19], the establishment of early embryo segmentation mechanisms and the transition from short-to-long-germ band mode of embryogenesis [20–24], the basal organisation of the head [25, 26], or the origin of wings and the capacity to fly (an issue that is currently hotly debated [27–35]). Overall, these examples point to important questions that are still open, ultimately revealing the need for establishing new model systems, in particular around the nodes of the tree where these key novelties/adaptations originated.

The advent of next-generation sequencing (NGS) techniques and new genome-editing technologies allow the re-examination of long-standing questions in evo-devo using comparative approaches. However, one of the challenges is getting access to the biological material, especially at the desired developmental stage for a particular study. Thus, there is a great interest in increasing the number of emergent model organisms that due to their key phylogenetic position or their specific traits would permit evo-devo studies in the precise clade of interest. Here, to contribute in this direction, we developed an Ephemeroptera laboratory model, *Cloeon dipterum.*

**Fig. 1:**
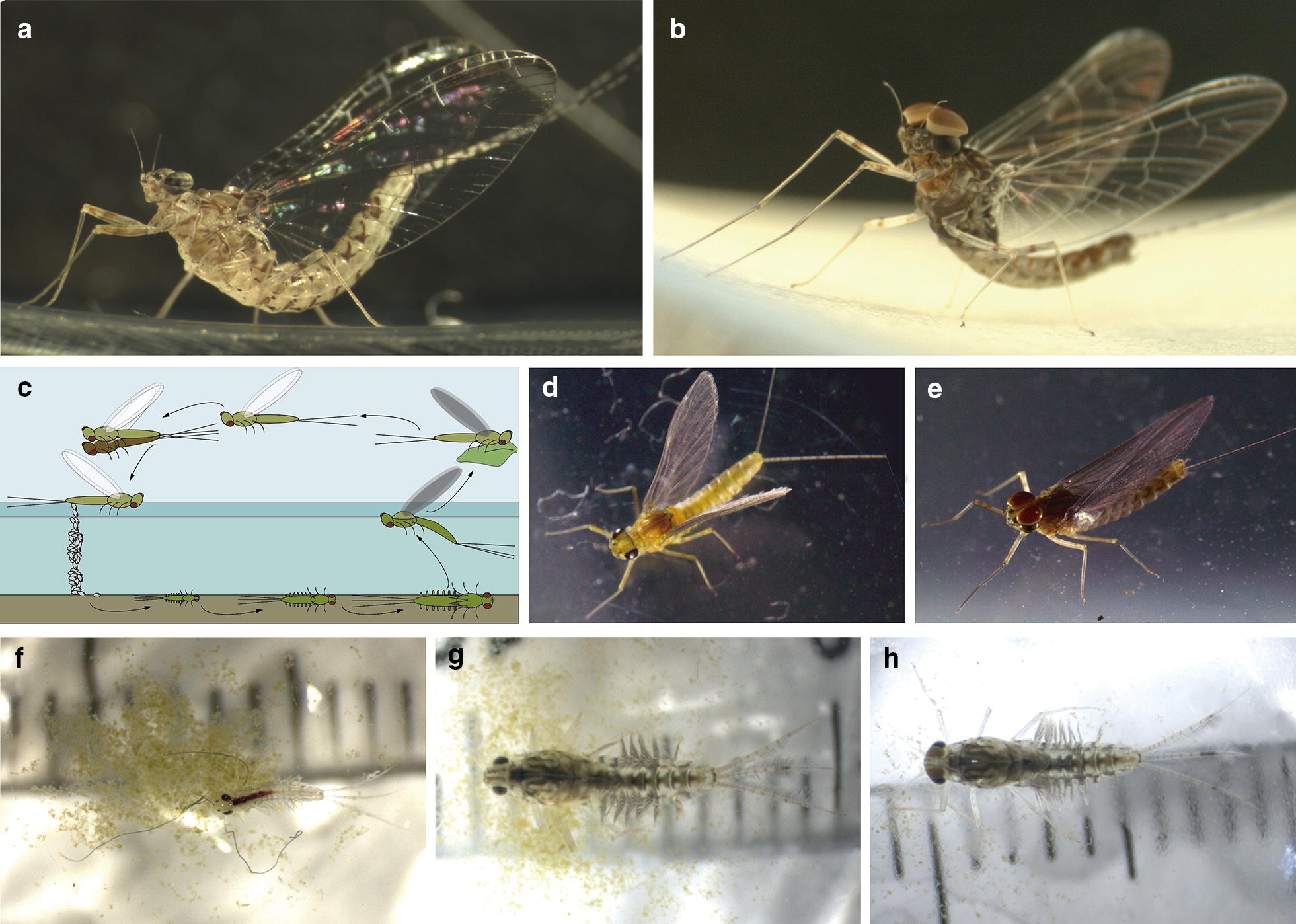
*C. dipterum* life cycle. **a** *C. dipterum* adult female, **b** *C. dipterum* adult male. **c** Cartoon depicting *C. dipterum* life cycle. Female lays the eggs in a water stream where they hatch as juvenile nymphs. After several moults nymphs emerge from the water to the land as immature subimagos. Then, they moult again to become sexually mature individuals that fly forming swarms to mate. **d** Female subimago. **e** Male subimago. **f** Early-mid nymph. **g** Late female nymph. **h** Late male nymph

Ephemeroptera (mayflies) is an order of winged hemimetabola insects that live in freshwater ecosystems. The Ephemeroptera order has over 3000 species distributed in 40 different families approximately [36, 37]. Mayflies belong to an ancient group of insects that were present already in the late Carboniferous or early Permian period [1]. Mayflies have a life cycle that consists of two well-defined phases. The aquatic phase that comprises embryogenesis and nymphal stages and the terrestrial phase, which consists on a sexually immature subimago and a sexually active imago (Fig. 1). Their aquatic phase makes mayflies ideal as bioindicators of the quality of freshwater ecosystems [38–40], while their terrestrial phase contributes to population dispersal; thus, mayflies have been used to investigate biogeographical events, such as dispersion and colonisation of new communities [41–43]. The duration of embryogenesis is variable, ranging from days to months, depending on the species and environmental factors, as the temperature [44]. Although the life cycle of some species, like *Baetis vernus*, *Ephemerella ignita* and *Ephoron shigae,* includes a programmed egg-dormancy phase or diapause [45–48], this is not the case for *C. dipterum*. Once the nymphs eclode from the eggs, they undergo a series of moults to finally moult into a terrestrial subimago that leaves the water. This sexually immature individual has to moult once more to become the sexually mature imago. This two-step moulting into the sexually mature individual is a striking singularity of mayflies [49, 50]. The mating occurs in flying swarms formed by hundreds of individuals several metres above the ground/water surface level [51–53].

In most phylogenetic analyses, Ephemeroptera is grouped together with Odonata (damselflies and dragonflies) as the sister group of Neoptera, the rest of winged insects ([54] and references therein). Therefore, extant mayflies are a key order to test the hypotheses postulated for the wing origin in pterygote insects. Their position in the phylogenetic tree also makes them an essential group to investigate segmentation, head specification and other morphogenetic processes occurring in the embryo, beyond the classical insect models already used to address these problems (*Drosophila*, *Tribolium*, *Oncopeltus*). Moreover, their particular life cycle with an aquatic and a terrestrial period makes mayflies a relevant organism to examine different adaptations to the land, such as the evolution of the extra-embryonic layers and other metabolic and physiologic traits derived from this complex life cycle, such as the hormonal control, ecdysis and metabolic rates.

By having established *C. dipterum* in the laboratory, we have now unlimited access to all embryonic and postembryonic stages through the year, permitting the study of fundamental processes that originated for the first time in Ephemeroptera or that are specific to the extant members of this order. Moreover, the development of a series of genomic and transcriptomic resources will facilitate comparative analyses at the genome, transcriptome and epigenomic levels that can clarify the role of certain genes and regulatory networks in the origin of those novelties. Finally, the high regenerative capabilities of *C. dipterum* [55–57], together with its short life cycle (which lasts from 40 to 60 days on average), make this species a significant and very useful system to investigate the regeneration of non-embryonic tissues in insects.

### *C. dipterum* continuous culture in the laboratory

*Cloeon dipterum*, from the Baetidae family, is one of the few ovoviviparous ephemeropteran species: the female keeps the fertilised eggs inside the abdomen and only when they are ready to hatch, after 10–20 days, the female sets down onto the surface of a water stream or pond and lays the eggs that sink to the bottom ready to eclode. Just few seconds after the eggs are laid, the nymphs hatch [58].

Individual lines were established and maintained in the laboratory for multiple generations starting from single gravid females captured in Dos Hermanas (Sevilla, Spain) and Alfacar (Granada, Spain). In the laboratory, gravid females are kept in a petri dish with a wet filter paper to avoid their desiccation. After 13 days, the female is first placed on the surface of unchlorinated water in a beaker to let it lay the eggs, but the duration of embryogenesis is a bit variable, between 13 and 17 days. If the embryos are ready to hatch, females immediately spawn. However, in case she does not spawn within the first minute on the water, the female has to be brought back to the petri dish to avoid the laying of underdeveloped eggs. It is therefore advisable to try to induce the spawning during several days until reaching the appropriate moment when the embryos are fully developed. The number of eggs a female can lay depends mainly on its nutritional condition. In general, bigger females produce larger clutches. In the laboratory, the females tend to lay between one hundred and three hundred eggs per clutch. Shortly after delivering the eggs, the females die.

**Fig. 2:**
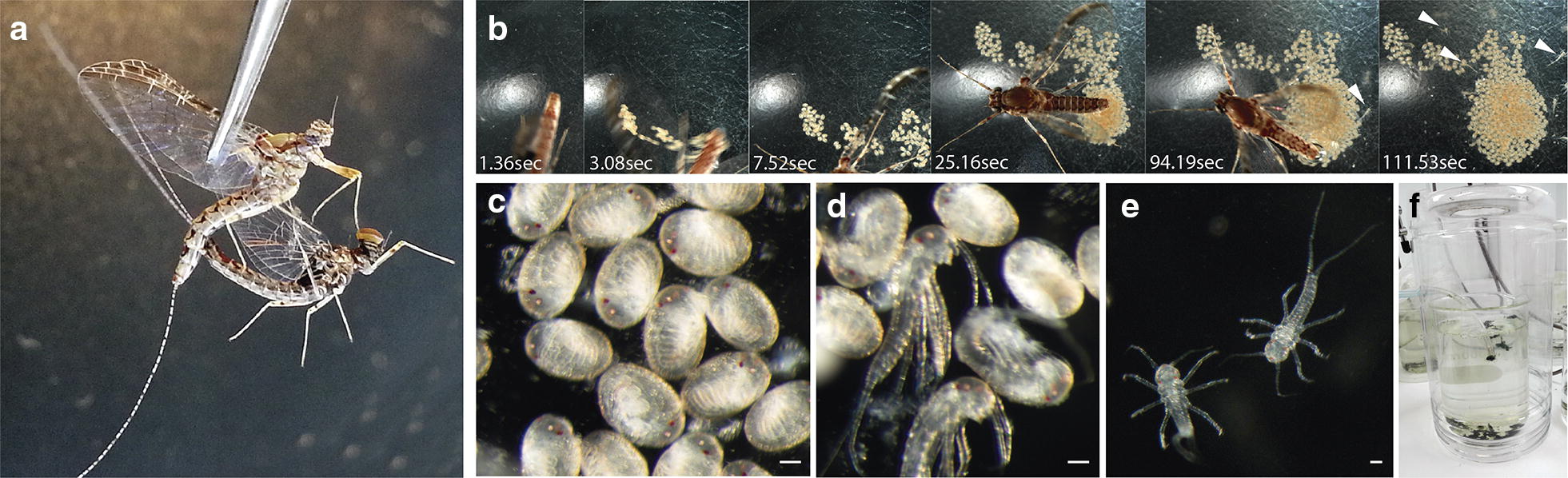
*C. dipterum* culture in the laboratory. a Couple of adults mating through forced copula. **b** Temporal sequence of a gravid female laying fertilised eggs that after 94 s hatch as swimming nymphs (white arrowheads). **c**, **d** Fertilised eggs and nymphs hatching. **e** Freshly hatched nymphs. **f** Culture system in the laboratory. Nymphs are in the beaker with bubbling water and algae. The beaker is placed inside a plastic bottle to keep the subimagos once they emerge from the water. Scale bars: 50 μm

The hatchlings take only a few seconds to hatch (Fig. 2b–e, Additional files 1 and 2) as swimming nymphs. They instantly start feeding on algae that are placed at the bottom of a 1000 ml beaker with approximate 700 ml of water. In the moment of hatching, the nymphs do not have external gills. It is only two moults later, approximately 72 h after hatching that seven pairs of gills are visible in the first seven abdominal segments. The nymphs are kept in the unchlorinated water in the beaker during the whole juvenile period. A portion of the water is replaced once a week, though the frequency can be increased if the culture becomes cloudy due to an excess of mayfly faeces or the overgrowth of algae. A bubbling tube connected to an air pump is introduced in the water to oxygenate it (Fig. 2f). The nymphs feed regularly on *Chara*, filamentous algae, pulverised vegetarian fish flakes or small pieces of carrot that are added to the water.

The beakers are placed inside 10 l plastic bottles (Fig. 2f), so when subimagos emerge from the last nymphal stage and leave the water, they remain inside the bottle and can be easily recovered. To avoid water condensation that could damage the newly emerged subimagos while they stay inside the plastic bottles, the plastic upper side is replaced by a small net. The subimagos are carefully collected and kept for 24 h in a tube with some wet paper to maintain the humidity and promote the last moult to imago, which happens some hours after the previous moult. To close the cycle in the laboratory, it is necessary to perform forced copulas [59], since mayflies mate during flight in large swarms [51–53]. To perform the mating, both male and female are grasped very carefully by the wings with forceps. The female is placed with the ventral side upwards and the most posterior region of the male is brought close to the female seventh abdominal segment. Males, then, clasp the abdomen of the female using their genital forceps or stylus, allowing the contact of the two external genitalia to engage the copula (Fig. 2a). Copulas have a variable duration: they can last from few seconds to several minutes. During this time, males bend themselves to favour the fertilisation of the eggs. After the copula, the male is discarded and the female is kept in a petri dish with a small piece of humidified filter paper. The culture is maintained in a 21-23 degrees Celsius room and a 12:12 light/dark illumination cycle.

### *C. dipterum* embryogenesis

**Fig. 3:**
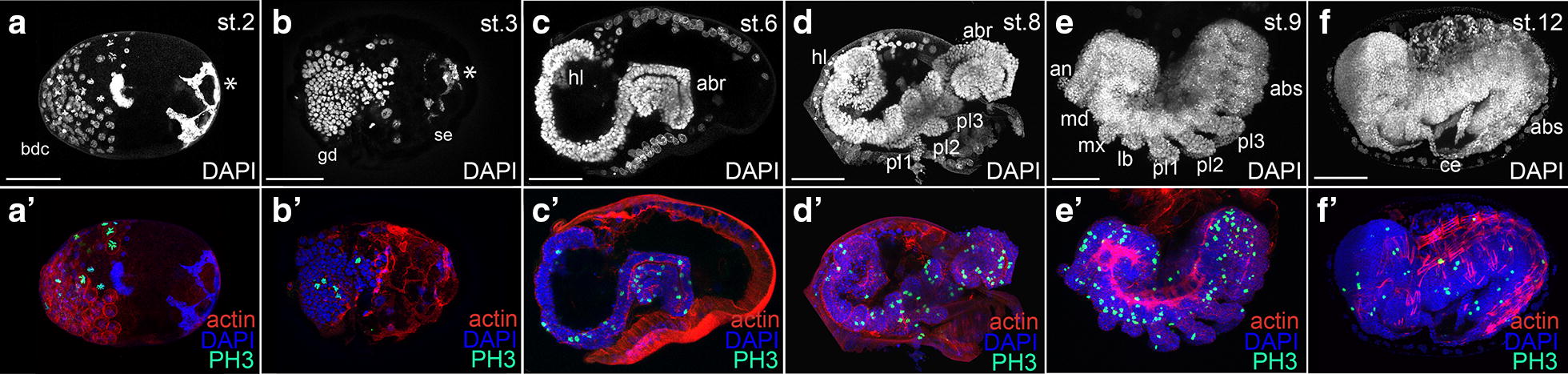
Representative phases of *C. dipterum* embryogenesis. Upper panels show embryo morphology detectable through DAPI staining (white). Lower panels show DAPI (nuclei, blue), Actin (cell contour cRed) and mitosis (anti-PH3, green). **a**–**a**′ Blastoderm formation (stage 2: st. 2) bcd: blastoderm cells are replicating, shown by PH3 staining (**a**′), in green. The asterisk highlights a DAPI-rich region located opposite to the embryo with unknown function. **b**–**b**′ Germ disc (gd) formation (st. 3). The asterisk highlights a DAPI-rich region located opposite the embryo which we have not identified. It disappears during subsequent stages. **c**–**c**′ S-shaped embryo (st. 6). The germ band elongates through active cell proliferation, shown by PH3 staining (abr: abdominal region; hl: head lobe). **d**–**d**′ Segmentation of the embryo (st. 8) starts from the cephalic (hl) and thoracic regions, which segments are already visible, towards the abdominal regions (abr). **e**–**e**′ Proctodaeum formation (st. 9). Segmentation progresses, appendages enlarge and get segmented (an: antenna, md: mandible, mx: maxilla, lb: labium, pl: pro-leg). **f**–**f**′ The abdominal regions are already segmented (abs: abdominal segments). Cercei (ce) are already visible. Dorsal closure proceeds. Scale bars: 50 μm

The establishment of the continuous culture of *C. dipterum* in the laboratory allows the study of the complete embryogenesis of these mayflies by obtaining the embryos directly from the abdomen of gravid females. Once the embryos are collected, it is possible to use antibodies and other markers to visualise the morphology of the embryo and morphogenetic processes occurring at specific developmental stages (Fig. 3).

*Cloeon dipterum* embryogenesis takes between 13 and 17 days, depending on the temperature. The morphogenesis in this species is similar to the previously described embryogenesis of other mayflies [60, 61]. Briefly, after egg cleavage the blastoderm is formed. Within the blastoderm, two populations of cells are soon distinguishable, the most posterior ones, with smaller nuclei that will form the germ disc and the larger and more anterior cells that will become the serosa (Fig. 3a, b). Thereafter, the germ disc starts elongating and the future cephalic region and future caudal segment addition zone become apparent. During the following highly proliferative stages, as showed by an increased density of PH3-positive mitotic cells (Fig. 3c′–e′), the embryo elongates within the egg, adopting a S-shape (Fig. 3c). As the embryo elongates, its most posterior region, which will correspond to abdominal segments, folds several times. After this phase, the embryo reaches its final length and its segmentation starts. Segmentation happens from anterior to posterior, with cephalic and thoracic appendages being the first to become visible (Fig. 3d). Afterwards, the embryo undergoes a series of final developmental events in which its final form is completed, including the appearance of the caudal filament, the two posterior cerci, the three ocelli and the compound eyes (Fig. 3e, f).

**Fig. 4:**
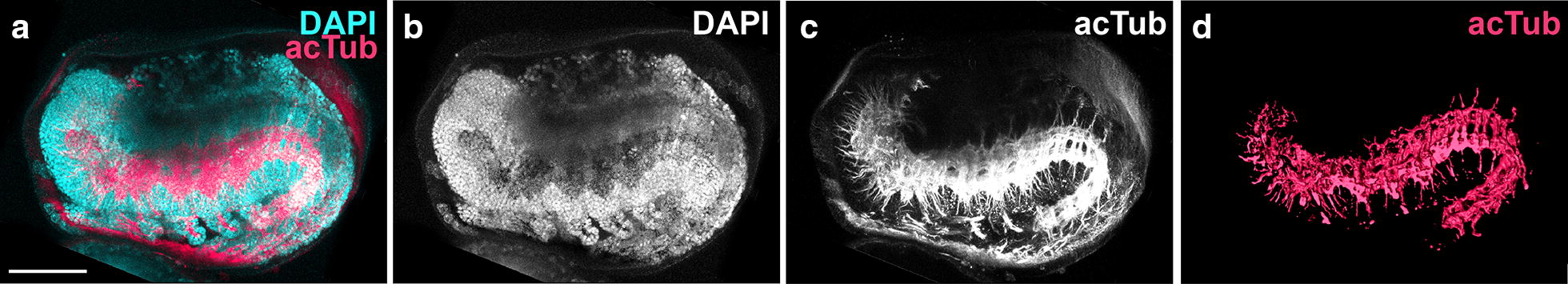
*C. dipterum* embryonic nervous system. **a**–**c** Embryo (DAPI staining reveals embryo morphology, **b** exhibiting the ventral nervous cord (staining using anti-acetylated alpha Tubulin antibody, **c**). **d** Surface reconstruction of the ventral nervous cord and its projections towards the appendages. Scale bars: 50 μm

**Fig. 5:**
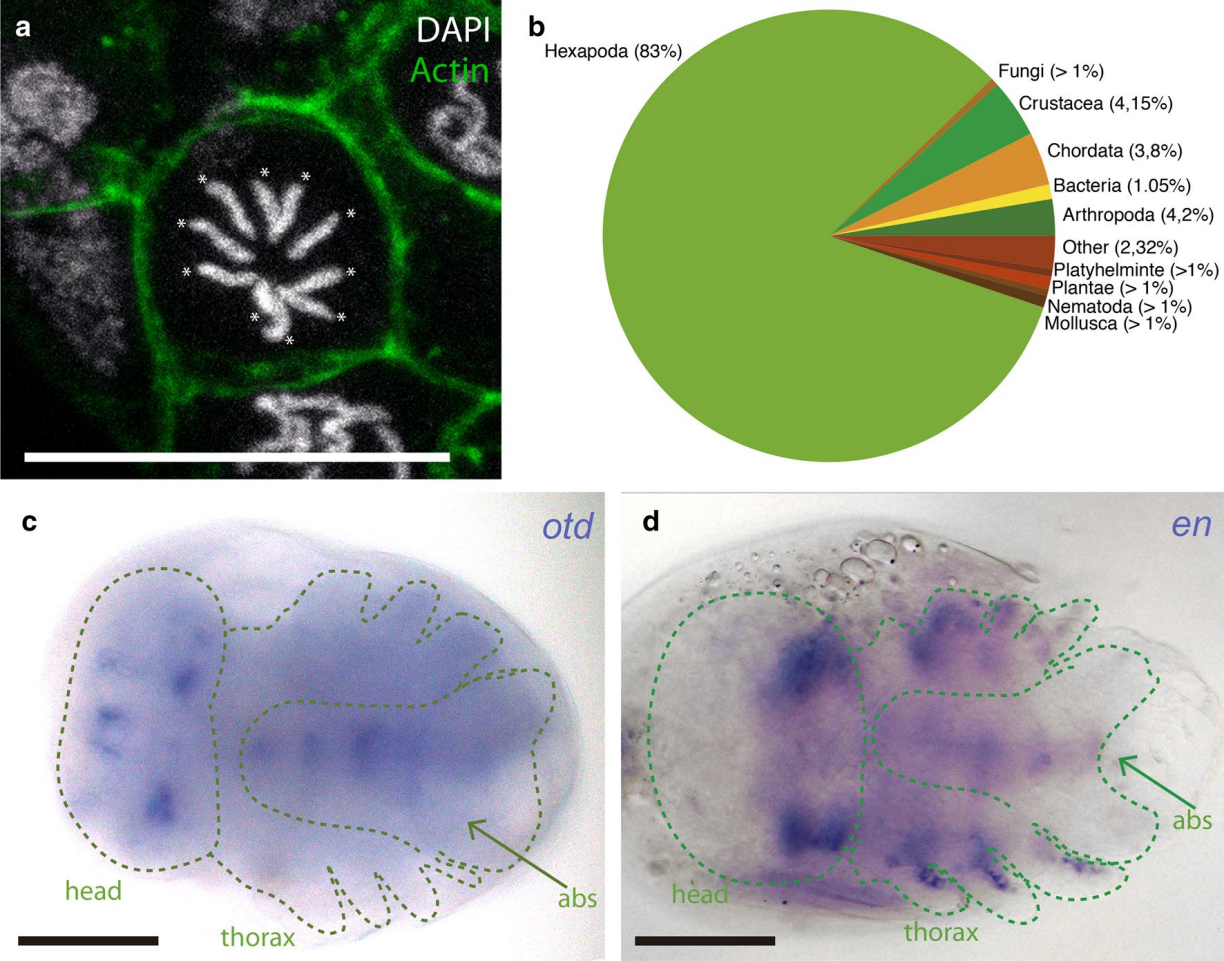
Genomics and transcriptomic tools. **a** The genome of *C. dipterum* is structured in a karyotype of 2*n* = 10 [95, 96]. Somatic embryonic cell showing condensed DNA in chromosomes (each of them highlighted with an asterisk). DNA stained with DAPI (white) and cell membrane visible through Phalloidin–Rhodamine staining (green). Scale bar: 20 μm. **b** Pie chart representing top BLASTp results of unigenes against UniRef90 protein database. **c** *otd* expression pattern in the mayfly embryo. **d** *en* expression domain in *C. dipterum* embryo

Beyond general morphology, access to all developmental stages also allows the study of the development of specific tissues and organs. We have tested protocols for immunofluorescence and in situ hybridisation (ISH) using non-radioactively labelled RNA probes to detect gene expression. We illustrate these protocols with the anti-acetylated Tubulin (acTub) antibody to mark axonal projection of the nervous system (Fig. 4) and with RNA probes against *orthodenticle* (*otd*) or *engrailed* (*en*) that define specific embryonic regions, such as the optic region and the segmental borders, respectively (Fig. 5c, d).

### The transcriptome of *C. dipterum*

Until now, there were no appropriate genomic tools available to investigate *C. dipterum* at the genetic level. Only a genomic survey sequencing for molecular markers to study *C. dipterum* population structure using 454 technology at low coverage has been reported [41, 62]. Therefore, to carry out the first characterisation of *C. dipterum* gene content, we sequenced the transcriptome of a male nymph. The assembly of the paired-end reads, using Trinity RNA-Seq de novo assembly software [63] resulted in 117233 transcripts. From these 117233 transcripts, we obtained 95053 peptide sequences using transDecoder [64] to get the longest translated ORFs. Running BLASTp, we got a list of 15799 sequences from UniRef90 database which showed homology to other sequences (*e*-value < 10*e*−6). These hits showed a majority of results, more than 80% (13059 best hits), within the hexapoda (insects and Collembola). The second most frequent groups of hits fell within Arthropoda (Chelicerata and Myriapoda, 4.24%) and Crustacea (4.15%) categories, which demonstrated the good quality of the assembly (Fig. 5b). Less frequent categories present in our best hit results corresponded, on the one hand, to bacteria and virus which probably derive from the mayfly microbiota, and on the other hand, Plantae and Red algae, which most likely belong to the gut content of the specimen at the moment of the RNA extraction, as *C. dipterum* feeds on algae and plants.

Although the transcriptome generated was obtained from a single male nymph, and it thus represents only the genes that are expressed at that particular developmental stage, it can nevertheless serve as a very useful resource to identify homologous transcripts and to design probes to perform subsequent expression pattern analyses of genes of interest expressed during nymphal stages and other stages, as in the case of *orthodenticle* (*otd*) and *engrailed* (*en*) (real time PCRs, in situ hybridisation, Fig. 5c, d).

This transcriptome assembly is a first step in order to have resources that can be used to tackle questions in the evolution of first winged insects at a genomic/transcriptomic level. Nevertheless, more tools are needed, so the high-quality genome sequencing project that is currently in progress will provide an invaluable resource and a platform for subsequent analyses (ATAC-Seq, Chip-Seq, etc.) to investigate long-standing questions related to the origin of pterygotes and other important traits that contributed to the diversification of insects.

### The regenerative potential of *C. dipterum*

The capacity to regenerate lost or damaged organs, body parts (or even whole organisms) is widespread throughout the animal kingdom [65–67]. Several phyla, such as Cnidaria, Platyhelminthes, Annelids, Arthropods or Vertebrates, have this ability. However, different species or even phyla have very different regenerative capabilities. For instance, Platyhelminthes (flatworms) use totipotent cells called neoblasts, to regenerate a complete organism from a few hundred cells [68–74], while other organisms, such as the crustacean *Parhyale*, rely on the dedifferentiation of cell populations to re-grow amputated limbs [66, 75, 76]. Despite the diversity of species that are able to regenerate and the varying modes, mechanisms and degrees of their regeneration capabilities, only a small number of organisms have been used to investigate how regeneration occurs. This is particular evident for insects, where only a handful of species have been used as models for regeneration studies [77], namely *Drosophila melanogaster*, which only regenerates undifferentiated primordia—the imaginal discs—and the gut [78–89], and two hemimetabolous insects, the cockroach *Blattella germanica* [90, 91] and the cricket *Gryllus bimaculatus*, which can take from 1 month to 18 weeks to regenerate an amputated limb [91–93].

**Fig. 6:**
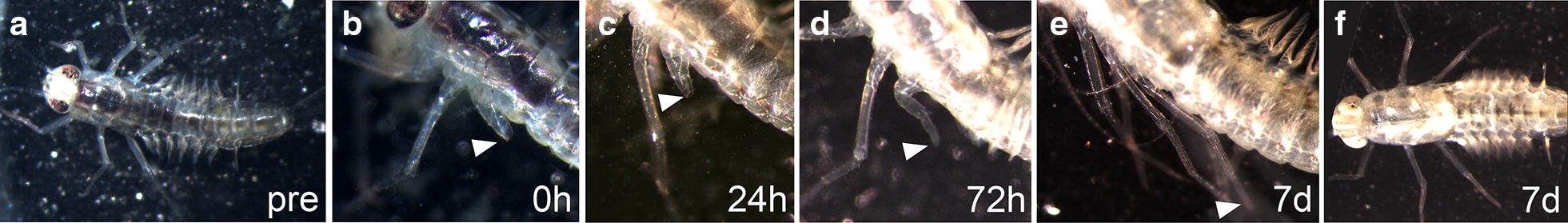
Leg regeneration of a *C. dipterum* nymph. **a** Mayfly nymph before amputation of the third leg. **b** Nymphal leg (white arrowhead) immediately after amputation. **c** 24 h after amputation the wound is healed (arrowhead). **d** 72 h after amputation, the tissue is already partially regenerated. **e**, **f** After 7 days, the amputated leg has recovered its initial size and shape with all the segments perfectly formed

Dewitz, already in 1890, described that mayfly nymphs were able to regenerate their gills completely after amputation [56]. Since then, several researchers [55, 57] confirmed these observations. Indeed, we observed that *C. dipterum* is able to regenerate gills, antennae, cerci and legs completely in a very short period of time, ranging from 6 to 9 days (Fig. 6). For instance, after amputation of the third pair of legs, *C. dipterum* takes less than 24 h to heal the wound and only 72 h to exhibit a clear re-growth of the appendage, completing the entire process in a period of no more than 7 days (Fig. 6). Thus, *C. dipterum* has extraordinarily rapid regenerative capabilities that could give this species a privileged status because of its fast regeneration of postembryonic, fully functional organs.

## Conclusions

Although Ephemeropterans have been the focus of biogeography, taxonomy and ecology studies, until now they have been very rarely used as a laboratory model to address developmental and evolutionary questions [35, 94], despite the fact they are fundamental to understand insect evolution at multiple time scales. Here, we present *C. dipterum* as an emergent model for evo-devo studies. There are several traits in this species that make it especially useful to answer long-standing questions in evolutionary biology. First, the setting up of a continuous culture system in the laboratory facilitates the access to all the developmental stages, and because of the ovoviviparism of *C. dipterum*, it allows having high numbers of synchronised embryos. The continuous culture also permits to obtain large amounts of material that can be used in genomics and transcriptomics assays. Second, the use of forced copulas ensures a complete control on the mating, so it is feasible to have inbred lines to reduce genetic heterozygosity which is necessary for genetic experiments or when applying functional genomics techniques. Moreover, the relatively short life cycle of *C. dipterum* permits the investigation of embryonic and postembryonic processes in a brief period of time and making experimental designs feasible. Although RNA interference techniques are not established in the mayfly yet, its aquatic life phase allows us to perform drug treatments, for example, to interfere with signalling pathways, by just adding the molecules to the water. However, these functional experiments are limited by the number of drugs available to alter specific gene networks, thus, efforts must be made to set up interference RNA and CRISPR/Cas9 methods to downregulate genes or edit the genome in *C. dipterum.*

Moreover, the generation of omic resources, through the sequencing of *C. dipterum* genome and different tissue- and stage-specific RNA-seq datasets, and the development of protocols to investigate changes in regulatory regions of the genome, such as ATAC-seq and ChIP-seq techniques, will provide a great resource to investigate evolutionary and developmental questions at the genomic level.

Beyond the technical and methodological advantages that *C. dipterum* confers, the key phylogenetic position, ecology, physiology and plasticity of mayflies make them an essential order to investigate very diverse topics, from genomic and morphogenetic events that occurred at the origin of winged insects, the origin of metamorphosis and hormone control of ecdysis, to the regenerative potential in insects.

## Additional files

**Additional file 1.** Female laying a clutch of embryos after being placed on the water surface.

**Additional file 2.** Nymphs hatching from the eggs few seconds after being laid.
